# Abraded optical fibre-based dynamic range force sensor for tissue palpation

**DOI:** 10.3389/frobt.2024.1489884

**Published:** 2024-11-11

**Authors:** Abu Bakar Dawood, Vamsi Krishna Chavali, Thomas Mack, Zhenyu Zhang, Hareesh Godaba, Martin Angelmahr, Kaspar Althoefer

**Affiliations:** ^1^ School of Engineering and Materials Science, Queen Mary University of London, London, United Kingdom; ^2^ Department of Fiber Optical Sensor Systems, Fraunhofer Heinrich Hertz Institute, Goslar, Germany; ^3^ Department of Engineering and Design, University of Sussex, Brighton, United Kingdom

**Keywords:** soft force sensor, dynamic range force sensor, optical sensing, fibre optic sensor, tissue palpation, minimally invasive surgery

## Abstract

Tactile information acquired through palpation plays a crucial role in relation to surface characterisation and tissue differentiation - an essential clinical requirement during surgery. In the case of Minimally Invasive Surgery, access is restricted, and tactile feedback available to surgeons is therefore reduced. This paper presents a novel stiffness controllable, dynamic force range sensor that can provide remote haptic feedback. The sensor has an abraded optical fibre integrated into a silicone dome. Forces applied to the dome change the curvature of the optical fibres, resulting in light attenuation. By changing the pressure within the dome and thereby adjusting the sensor’s stiffness, we are able to modify the force measurement range. Results from our experimental study demonstrate that increasing the pressure inside the dome increases the force range whilst decreasing force sensitivity. We show that the maximum force measured by our sensor prototype at 20 mm/min was 5.02 N, 6.70 N and 8.83 N for the applied pressures of 0 psi (0 kPa), 0.5 psi (3.45 kPa) and 1 psi (6.9 kPa), respectively. The sensor has also been tested to estimate the stiffness of 13 phantoms of different elastic moduli. Results show the elastic modulus sensing range of the proposed sensor to be from 8.58 to 165.32 kPa.

## 1 Introduction

Tissue palpation is a crucial technique used by clinicians to identify tissue abnormalities. This diagnostic procedure is performed by exerting force - using one’s fingers - to assess various aspects of tissue health including lumps, swelling and abnormalities in tissue texture or consistency (Konstantinova et al., 2017). In the case of Minimally Invasive Surgery (MIS), surgeons have restricted access to the tissues and organs. The resulting lack of direct tactile feedback, restricted visibility, and risk of tissue trauma, make it difficult for surgeons to assess the tissue characteristics (van der Putten et al., 2008).

Numerous solutions have been proposed to compensate for, or indeed replace the tactile aspect of haptic feedback by measuring the tissue stiffness. Most of these involve physical contact though a few non-contact solutions have also been proposed Konstantinova et al. (2014). The contact-based proposals use multiple sensing technologies including optical (Ahmadi et al., 2010; Xie et al., 2013), vibro-acoustic (Sühn et al., 2023), magnetic (Zhang et al., 2021) and pneumatic (Wanninayake et al., 2013). Kawahara et al. proposed a non-contact method of differentiating tissue stiffness (Kawahara et al., 2010). The system comprised of an air nozzle and an optical displacement sensor. A stream of air pressure through the nozzle would create a corresponding displacement signal. The system was tested on porcine lung tissue.

For tissue stiffness estimation using contact sensors, two principal approaches have been used. In one approach, surgical probes with integrated force and displacement sensors are calibrated for force and displacement, to estimate the stiffness of the tissue Kim et al. (2018), Kim et al. (2020), Xie et al. (2014). The other approach is to have indentation information for different levels of stiffness. This can either be achieved by changing the stiffness of the structure Herzig et al. (2018) or by comparing stiffnesses of multiple structures (Faragasso et al., 20144). A variable stiffness robotic probe has been designed by Herzig et al. (2018). It works on the principle of a variable lever mechanism which is realised by changing the active length of a rod connecting two links. A simulation study was also performed to investigate different design parameters for a specific stiffness range, relevant to a particular application. However, this probe was designed for external abdominal palpation (Herzig et al., 2018). Similarly, a stiffness estimation probe, developed by Faragasso et al. uses four indenters loaded by 2 springs of different stiffness (Faragasso et al., 2014). The displacement caused by these indenters is measured with an endoscopic camera such that the stiffness of the target material can be estimated. This complex design had certain limitations caused by the friction and the parallel mechanism of the springs.

Typically, force sensors have a predetermined sensing range and sensitivity level - indeed only a few attempts have been made to develop sensors with adjustable force range and sensitivity. Raitt et al. (2022) developed a stiffness controllable sensing tip that uses a camera to observe the deformation of a silicone membrane. The membrane stiffness can be controlled by pneumatic pressure, resulting in an adjustable force range. The maximum force measured using this sensor was 2.799N making it unsuitable for palpation as the forces applied during palpation range from 4 to 6N (Konstantinova et al., 2017). Another dynamic force range sensor, ESPRESS.0, developed by Jenkinson et al. also uses pneumatic pressure to adjust the stiffness of the membrane. The membrane is connected to a number of coloured liquid filled tubes and the pressure inside these tubes is adjusted using air from a syringe pump. The force is measured with a camera, by tracking the menisci of fluid in all tubings (Jenkinson et al., 2023). In relation to MIS, palpation sensors need to be disposable, and therefore have simpler design - something that ESPRESS.0, on account of its design complexity and fabrication, is not.

**FIGURE 1:**
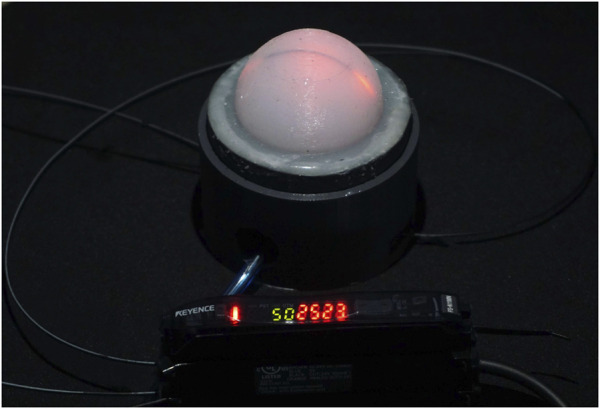
Proposed abraded optical fibre-based dynamic range force sensor. The optoelectronic system (Keyence) and a pneumatic inlet can be seen in the figure.

In this paper we present a novel, soft, low-cost, dynamic range sensor based on abraded optical fibre, as shown in Figure 1. The deformation of the abraded optical fibre within the silicone dome can be calibrated to the applied force, and the chamber within the dome can be pressurised to adjust its stiffness, and in turn, the force range and sensitivity of the sensor.

This paper’s contributions are:

1. Development of a novel and disposable force sensor using abraded optical fibre;
2. Realisation of a dynamic force range and variable stiffness by controlling the sensor’s internal pressure;
3. Estimation of elastic moduli by training a multivariate polynomial model on data from 13 phantoms;

## 2 Materials and methods

### 2.1 Principle

Abraded optical fibres have successfully been employed in bending sensors (Godaba et al., 2020). The bending of abraded optical fibres causes attenuation of transmitted light, such that by monitoring the light attenuation transmitted through the fibre to the receiver, one can estimate the fibre curvature.

**FIGURE 2:**
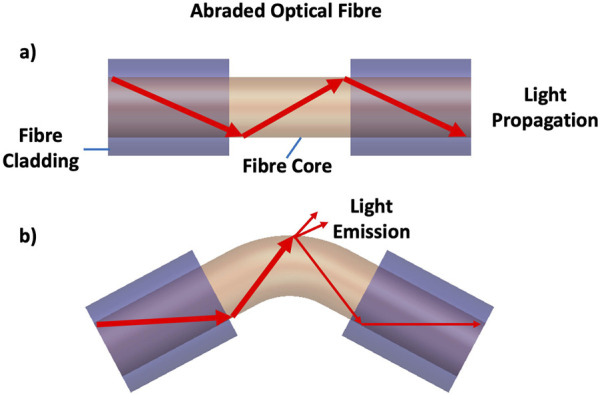
An abraded optical fibre as a bending sensor. **(A)** shows the optical fibre with removed jacket and sanded cladding. **(B)** shows light escaping through the abraded cladding when the optical fibre is bent.

All optical fibres display some attenuation of transmitted light when bent. However, in a standard, commercial optical fibre, this light loss is minimal and only small attenuation is experienced at very high curvatures. To use an optical fibre in a bending sensor, the sheath is stripped off and the cladding is removed, such as by sanding the fibre surface. This procedure increases the amount of light that scatters out from the fibre core when the fibre is bent, resulting in a significant reduction in the transmitted optical signal intensity. This is shown in Figure 2.

**FIGURE 3:**
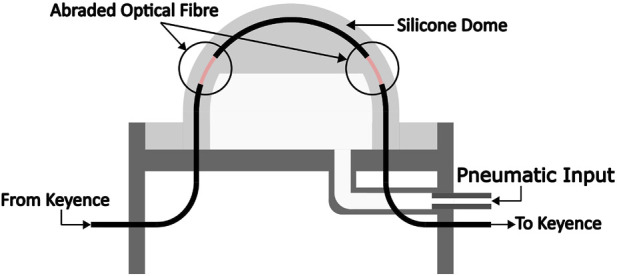
The cross-sectional view of an abraded optical fibre-based dynamic force range sensor. An inlet for variable pneumatic pressure can also be seen in the figure.

Light attenuation in abraded optical fibres in response to bending can also be used to measure force. Figure 3 shows the cross-sectional view of our proposed design. The optical fibre integrated into the silicone dome has been abraded in two specific areas as shown. The mechanism works as follows: when a force is applied to the top of the dome, it undergoes deformation and moves downwards; this increases the curvature of the two abraded regions, resulting in a decrease in light intensity transmitted through the fibre. In contrast to designs using abraded region at the centre of the dome, our two abraded sections near the sides of the dome allow for changes in light intensity across a large compression range, thereby increasing the force sensing range.

### 2.2 Design and fabrication

The design and the internal components of the sensor are shown in the cross-sectional view of Figure 3.

First, a two piece mould for the hollow silicone dome (40 mm diameter and 2.5 mm wall thickness thickness) was designed and 3D printed using PLA (Polylactic Acid) filaments on a commercial 3D printer (Ultimaker S3). Then the optical fibre (Mitsubishi Eska) with an outer diameter of 1 mm and core diameter of 0.5 mm, was stripped and abraded in two 10 mm sections. The abraded optical fibre was held in place in the mould by passing it through pre-printed holes, before closing it up with four M5 screws. Parts A and B of curable silicone EcoFlex 00-50 (Smooth-On, Inc.) were mixed in equal parts and degassed to remove any entrapped air bubbles. EcoFlex was then injected into the mould, degassed for a second time, and then cured at room temperature. The dome was designed with a thicker flat region between the abraded sections. This modification concentrates the deformation in the abraded areas of the fibres, leading to an increase in curvature.

Once cured, the silicone dome with the integrated abraded optical fibre was taken out of the mould. A base for the silicone dome was then 3D printed, also using PLA. This base had two holes through which to connect each end of the abraded optical fibre with the opto-electronic circuitry. A pneumatic inlet was designed to vary the pressure within the silicone dome, which was affixed to the base using silicone glue (Silpoxy by Smooth-On, Inc.) and tested to ensure there was no air leakage.

### 2.3 Experimental setup

**FIGURE 4:**
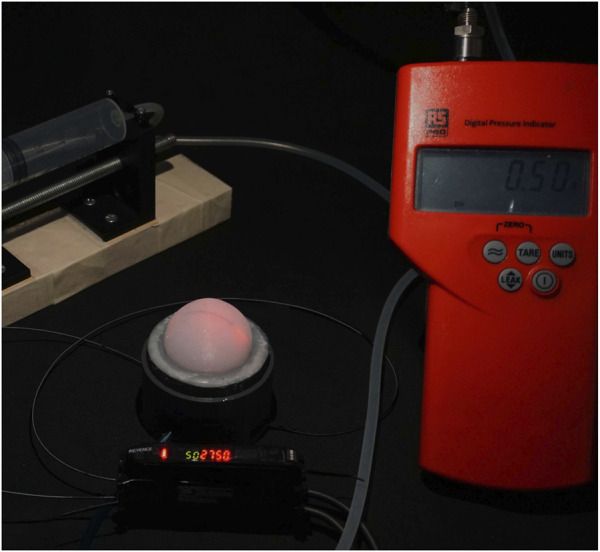
The experimental setup showing the sensor and the lead screw-driven syringe pump to create pneumatic pressure. The output of syringe pump is connected to a digital pressure indicator and to the sensor.

The experimental setup consisted of two systems - one pneumatic and one optical. The air pressure was generated by using a lead screw-based injection pump. The lead screw was coupled with a stepper motor controlled by an Arduino. The air pressure inside the sensor was manually controlled, using an RS Component digital pressure indicator as feedback. An opto-electronic system (Keyence sensor, FS-N11MN) was used to measure the light intensity from the abraded optical fibre. A Keyence opto-electronic system emits and receives light through the optical fibre and measures the change in the light intensity. These sensors emit light of a certain wavelength (630 nm) and have the added advantage of optical isolation which means that the ambient light doesn’t affect the sensor reading. The Keyence opto-electronic converts the change in light intensity to a voltage (0-5 V) and this voltage is recorded using an Analogue to Digital Converter (ADC) of an Arduino Uno micro-controller. The complete experimental setup is shown in the Figure 4.

**FIGURE 5:**
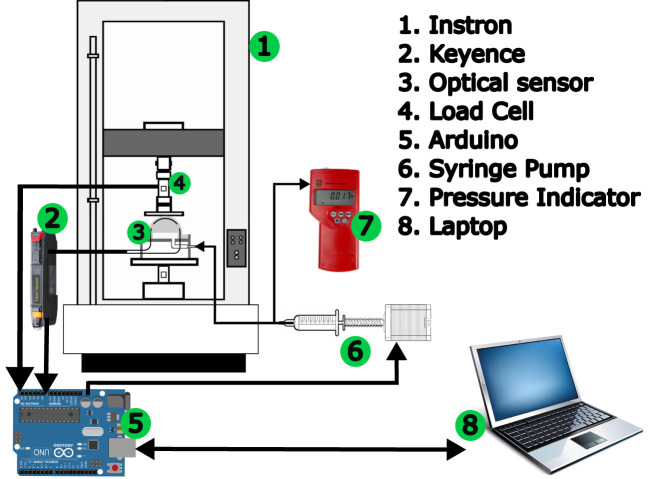
This diagram illustrates the connections of various components for experiment. Optical sensor is our proposed sensor being tested.

To calibrate the sensor for force, we used a universal testing machine, the Instron 5900, equipped with a 100 N load cell. For the experiments, a flat circular indenter with a 60 mm diameter was 3D printed using PLA (Polylactic Acid) and mounted on the moving head of the universal testing machine. The indentation depth was set at 6 mm for all experiments, as deeper indentation affects the vertical sections of the fiber. Rather than merely deflecting the middle section, higher indentations could increase the risk of fiber fracture. The displacement and force data from the Instron 5900 was recorded using analogue outputs. These were also connected to the Arduino. A Python code was written to enable communication with the laptop and Arduino, and data was recorded in. csv files. The data acquisition rate was calculated to be 60 Hz (60 data samples per second). Figure 5 shows the flowchart illustrating the connections of various components during experimentation.

Force sensing response and the dynamic force range for different dome pressures, are evaluated through a series of experiments. The sensor is cyclically loaded to 6 mm compression using the 3D printed indenter at two different displacement rates of 5 mm/min and 20 mm/min, these two rates chosen in order to observe the rate-dependent hysteresis in the sensor. To understand the influence of internal dome pressure on the dynamic force range of the sensor, these cyclic load/unload tests at each displacement rate, were conducted for internal dome pressures of 0 psi (0 kPa), 0.5 psi (3.45 kPa) and 1 psi (6.9 kPa). Five samples of each set-up were recorded.

The sensor was also subjected to repeatability tests, involving ten cycles of 6 mm indentations at the higher indenter displacement rate of 100 mm/min for each of the three set pressures (0 psi, 0.5 psi and 1 psi).

## 3 Results and discussion

This section is divided into three subsections. First we curve fit the experimental data and discuss the performance followed by the calculation of maximum percentage hysteresis. Then we discuss how the stiffness and sensitivity of the sensor are related to internal pressure. Finally, we use curve fitting to calculate the force and compare it with the ground truth.

### 3.1 Loading and unloading

**FIGURE 6:**
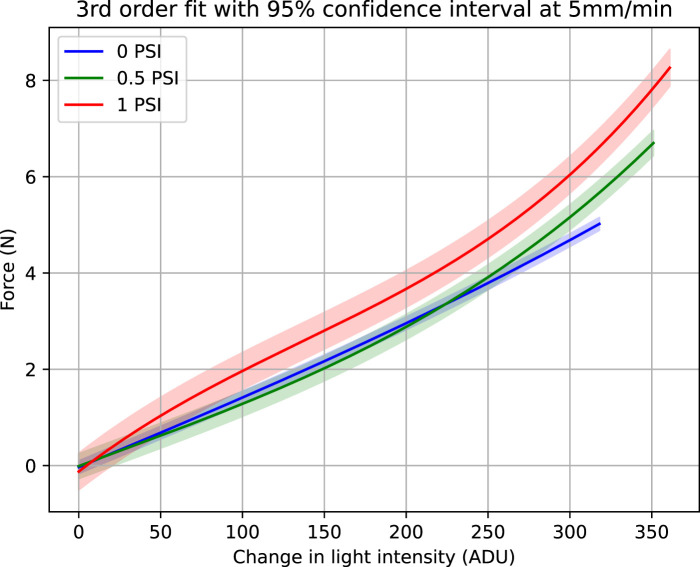
Force vs. light intensity change at 5 mm/min. Figure shows third order fit with 95% confidence intervals at three internal pressures, 0, 0.5 and 1 PSI.

**FIGURE 7:**
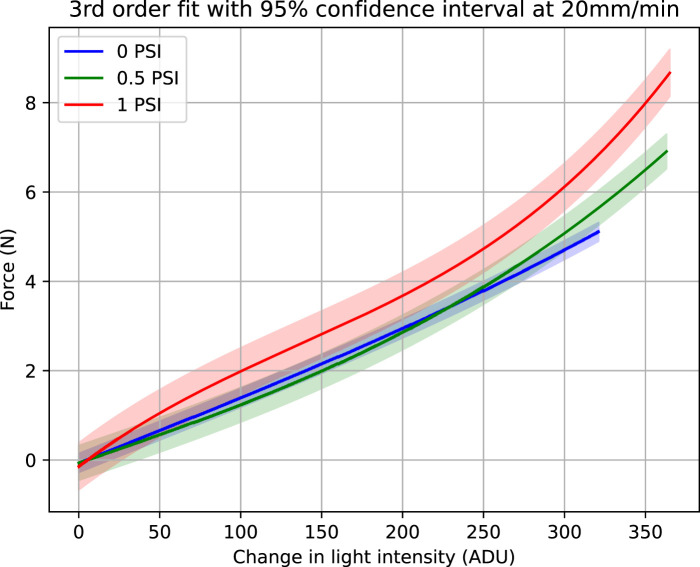
Force vs. light intensity change at 20 mm/min. Figure shows third order fit with 95% confidence intervals at three internal pressures, 0, 0.5 and 1 PSI.

As discussed in the previous section, each experiment was repeated five times. At each internal pressure, the loading and unloading data of these five experiments was fitted with third order polynomial. This polynomial fitting is plotted with 95% confidence interval and is shown in Figures 6, 7.

Both the Figures 6, 7 show that with increased pressure the force range of the sensor also increases while its sensitivity decreases. Although third order fit has been used, each light intensity change corresponds to only a single value of force. By comparing the two Figures 6, 7, it is obvious that the 95% confidence interval band widens when the indentation rate is higher. The goodness of the fit and the variability of the data was analyzed through regression analysis. Adjusted R^2^, Root Mean Square Error (RMSE) in Newtons are calculated for the performance evaluation of third order polynomial fitting at 5 mm/min and 20 mm/min. These evaluations are shown in Tables 1, 2, respectively.

**TABLE 1:** Force sensing curve fitting performance evaluation at 5 mm/min.

	Adjusted R^2^	RMSE (N)
0 PSI	0.9979	0.0668
0.5 PSI	0.9952	0.1313
1 PSI	0.9932	0.1933

**TABLE 2:** Force sensing curve fitting performance evaluation at 20 mm/min.

	Adjusted R^2^	RMSE (N)
0 PSI	0.9953	0.1064
0.5 PSI	0.9906	0.1967
1 PSI	0.9890	0.2671

Another interesting observation in Figures 6, 7 relates to the saturation of the sensor data. At lower internal pressure values, the sensor response does not show any saturation against measured force. However, when higher pressure is applied, sensitivity starts decreasing as the force range increases. This phenomenon is evident when 1 psi is applied and the sensor response starts saturating.

This can be explained by considering the change in stiffness of the sensor. At lower pressure the compliance of the silicone dome and the optical fibres mitigates the saturation phase of the sensor response. However, at elevated pressures, the stiffness of the silicone dome increases, resulting in sensor response saturation.

Comparison of Figures 6, 7 suggests that hysteresis increases for higher indentation rates (Mukashev et al., 2022). By comparing the maximum force measured during saturation for 6 mm indentations, we observe that the force achieved at a rate of 20 mm/min is higher than when using the lower 5 mm/min rate. This is because at a higher strain rate, greater force is required to create the same amount of deformation (Tatiraju and Han., 2010; Kenry et al., 2015).

The maximum percentage errors calculated for the hysteresis at two indentation rates of 5 mm/min and 20 mm/min are shown in Table 3. Similarly, Table 4 shows the maximum force being measured at each pressure and indentation rates.

**TABLE 3:** Maximum percentage errors at two indentation rates.

	0 PSI	0.5 PSI	1 PSI
5 mm/min	1.88%	3.72%	3.48%
20 mm/min	3.19%	6.17%	4.44%

**TABLE 4:** Maximum force measured during the experimentation.

Displacement Rate (mm/min)	0 PSI		0.5 PSI		1 PSI	
	Mean force (N)	S.D. (N)	Mean force(N)	S.D. (N)	Mean force(N)	S.D. (N)
5 mm/min	4.91	± 0.008	6.54	± 0.017	7.91	± 0.137
20 mm/min	5.02	± 0.004	6.70	± 0.027	8.83	± 0.238

**FIGURE 8:**
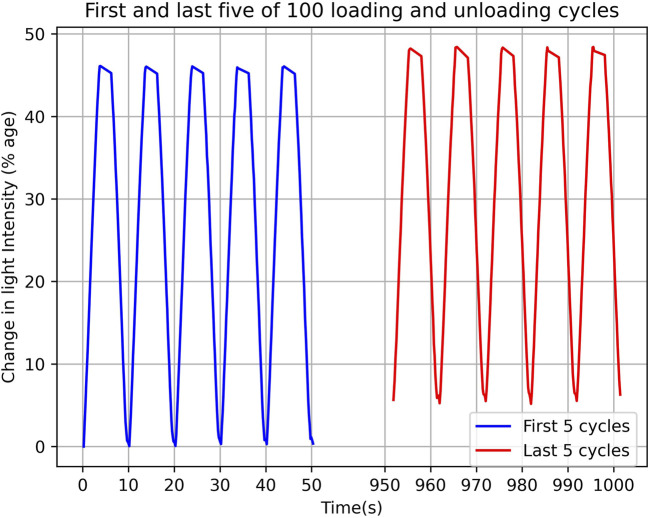
The first and last five cycles of 100 loading and unloading cycles. Figure shows the absolute percentage change in light intensity with respect to time.

To investigate the effect of repeated loading and unloading cycles on sensor response, a repeatability test was conducted at an internal pressure of 0 PSI. During this test, the sensor was indented to a depth of 6 mm at a feed rate of 100 mm/min. The indentation was maintained for two seconds, with no time delay between cycles. A total of 105 samples were tested, with the first five samples excluded from the analysis. Figure 8 presents the first and last five samples from the remaining 100 cycles.

During the repeatability experiment, a drift in the sensor response was observed. This phenomenon can be attributed to the viscoelastic relaxation behaviour of silicone materials. To reduce this effect, sufficient relaxation time between each cycle is typically required. However, the objective of this experiment was to push the sensor to its limits by applying maximum indentation at a high rate, with no relaxation time between cycles. The observed drift in the sensor’s zero state and indented state was calculated to be 5.53% and 4.28%, respectively.

### 3.2 Stiffness and sensitivity

The stiffness of the sensor can be calculated by using Hooke’s law that states,

F=kx (1)

where, F is the applied force, k is the stiffness of the spring and x is the displacement. Stiffness was therefore calculated using the force measured by the Instron load cell and the displacement of the indenter. Values for sensor stiffness under different pressures are shown in Table 5. These values were calculated using five samples of the data for a slow indentation rate, i.e. 5 mm/min.

**TABLE 5:** Sensor stiffness at different applied pressures.

	0 PSI	0.5 PSI	1 PSI
Mean (N/mm)	0.808	1.072	1.314
Standard Deviation	± 0.0035	± 0.0029	± 0.0046

The sensitivity of a sensor is defined as,

$$Sensitivity=\frac{\Delta Output}{\Delta Input}$$ (2)

where Δ Output is the change in output - Analogue-Digital-Units in our case - and Δ Input is the change in force. Values for sensitivity under different pressures are shown in Table 6. These values were also calculated using five samples of the data for slow indentation rate, i.e. 5 mm/min.

**TABLE 6:** Sensor sensitivity at different applied pressures.

	0 PSI	0.5 PSI	1 PSI
Mean (ADU/N)	64.645	53.949	46.110
Standard Deviation	± 0.561	± 0.486	± 0.28

Notably, the values for stiffness and sensitivity were calculated under the assumption of the sensor’s linear behaviour. Non-linear response would result in values that vary along the force-displacement and ADU-force curves of the sensor.

**FIGURE 9:**
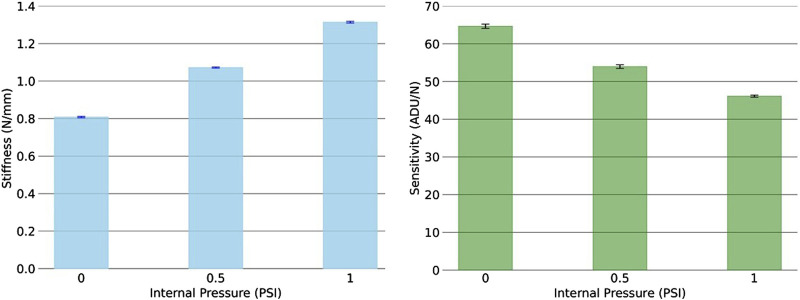
Stiffness and sensitivity with different internal pressure. The plots demonstrate that with an increase in internal pressure, stiffness of the sensor increases however, the sensitivity decreases.

The results presented in Tables 5, 6 have also been plotted and shown in the Figure 9.

### 3.3 Force estimation

The accuracy of force estimation was assessed by comparing the repeatability test data with the third order polynomial equations. Root Mean Square Error (RMSE) and R^2^ values were calculated at each internal pressure.

**FIGURE 10:**
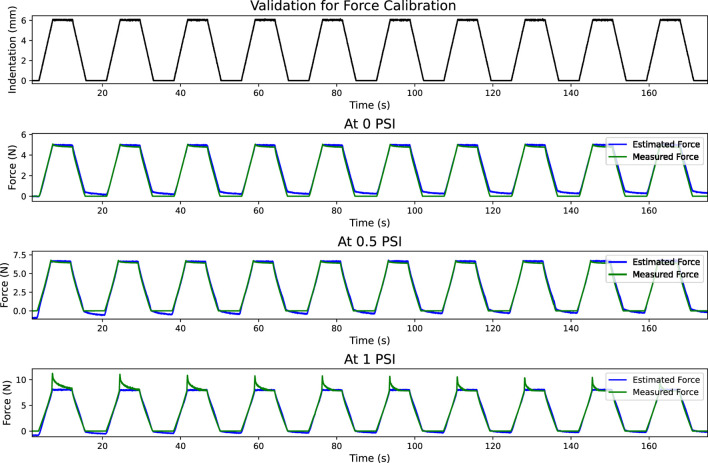
Validation for the calibrated force for 10 loading and unloading cycles. The first plot shows the indentation. Measured and estimated forces are shown in the remaining plots.

Figure 10 shows the sensor response for ten loading/unloading cycles at three different applied pressures using an indentation rate of 100 mm/min. Curve fitting equations were used to estimate the force from the sensor data. Measured force and estimated force are shown in subplots for each applied pressure. We can see that at higher inflation pressure, when the indenter reaches maximum compression, the actual force reduces with time while the estimated force value is nearly constant. This is because the optical fibre sensor reading is based on the deformation of the fibre, which remains constant during this period in which the indenter is not moving. However, silicone rubber is a viscoelastic material and undergoes stress relaxation. Due to this property of the material, the actual force reduces with time. The first subplot shows the indentation profile. These performance metrics have been presented in Table 7.

**TABLE 7:** Force sensing evaluation parameters.

	0 PSI	0.5 PSI	1 PSI
RMSE (N)	0.273	0.288	0.457
R^2^	0.982	0.989	0.983

It is to be noted here that the cyclic loading/unloading tests were performed at an indentation rate of 100 mm/min. Results show that force estimation at 0 psi is precise, with minimal drift. At higher pressures, however, the error increases due to hysteresis.

### 3.4 Elastic modulus estimation

The experiments presented in this section aim to assess the effectiveness of the proposed sensor in estimating the stiffness of various materials. The section is divided into two parts: the first addresses the development of the material phantoms, while the second focuses on estimating their Modulus of Elasticity.

#### 3.4.1 Phantom development

To assess the sensor’s performance in estimating tissue stiffness, multiple phantoms were fabricated according to the experimental protocols outlined by Raitt et al. (2022). These phantoms were designed to cover the range of elastic moduli of numerous healthy human tissues. The materials used for their fabrication were from the Ecoflex series by Smooth-On. Ecoflex 00-10, Ecoflex 00-30, and Ecoflex 00-50, along with Smooth-On thinner were used to achieve phantoms with varying stiffness levels. The silicone mixtures were thoroughly mixed, degassed, and poured into molds to produce cylindrical phantoms measuring 50 mm in diameter and 30 mm in height. Each phantom underwent compressive testing using a 1 kN load cell on the Instron 5900 machine. The phantoms were preloaded to 0.1 N and then subjected to a compressive strain of 20 mm (66.6%). Each phantom was tested 12 times, with six repetitions on each side; the first repetition on each side was excluded, leaving 10 valid samples per phantom. The elastic modulus was calculated using the data from the initial 3 mm (10%) of compression. The mixing ratios and the calculated elastic moduli of the phantoms are presented in Table 8.

**TABLE 8:** Phantoms’ material composition and elastic moduli.

Sr #	Material composition	Mean elastic modulus (kPa)	Standard deviation
1	Ecoflex 00-50 (A+ B+ 0% T)	165.32	± 4.07
2	Ecoflex 00-50 (A+ B+ 12.5% T)	119.64	± 4.42
3	Ecoflex 00-30 (A+ B+ 0% T)	102.79	± 4.56
4	Ecoflex 00-30 (A+ B+ 12.5% T)	72.67	± 3.12
5	Ecoflex 00-10 (A+ B+ 0% T)	57.22	± 1.65
6	Ecoflex 00-10 (A+ B+ 12.5% T)	38.23	± 1.65
7	Ecoflex 00-10 (A+ B+ 25% T)	28.19	± 0.74
8	Ecoflex 00-10 (A+ B+ 37.5% T)	20.06	± 0.60
9	Ecoflex 00-10 (A+ B+ 50% T)	16.19	± 0.38
10	Ecoflex 00-10 (A+ B+ 62.5% T)	12.74	± 0.56
11	Ecoflex 00-10 (A+ B+ 75% T)	9.39	± 0.11
12	Ecoflex 00-10 (A+ B+ 87.5% T)	8.56	± 0.08
13	Ecoflex 00-10 (A+ B+ 100% T)	7.00	± 0.11

#### 3.4.2 Elasticity estimation methodology

Following the characterization of the phantoms, an experiment was conducted to evaluate the sensor’s response when indenting phantoms of varying stiffness. The sensor was indented into each phantom to a depth of 6 mm at a rate of 20 mm/min. Three internal pressure levels 0 PSI, 0.5 PSI, and 1 PSI were selected, as used in previous experiments, and each condition was repeated five times. In total, the experiment involved 13 phantoms, 3 pressure levels, and 5 repetitions, resulting in 195 samples. After data collection, the ADC values were converted into percentage changes in light intensity across the entire dataset. The maximum percentage change for each of the 195 samples was extracted and organized by phantom. For each phantom, this process yielded five samples, each characterized by three features: the maximum percentage change at 0, 0.5, and 1 PSI, along with a target variable representing the elastic modulus of the phantom. The dataset was then divided into training and testing sets in a 3:2 ratio, ensuring that all phantom samples were split uniformly. A third-degree multivariate polynomial regression model was trained using the training data. Subsequently, the model’s performance was assessed using the test data, with the root mean square error (RMSE) calculated in both kPa and percentage terms as shown in Table 9.

**TABLE 9:** Stiffness estimation accuracy.

Sr #	1	2	3	4	5	6	7
Elastic Modulus (kPa)	165.32	119.64	102.79	72.67	57.22	38.23	28.19
RMSE (kPa)	15.44	8.69	4.39	1.12	4.07	2.67	0.28
RMSE (%)	9.34	7.26	4.27	1.55	7.12	6.98	0.98

Sr #	8	9	10	11	12	13
Elastic Modulus (kPa)	20.06	16.19	12.74	9.39	8.56	7.00
RMSE (kPa)	1.32	0.06	0.51	1.20	0.48	1.77
RMSE (%)	6.58	0.34	3.97	12.77	5.62	25.24

Table 5 shows the estimated elastic modulus of 13 phantoms with an average RMSE of 3.23 kPa. The table also indicates that the RMSE for the phantom with an elastic modulus of 7.00 kPa is considerably higher than that of the other phantoms. This discrepancy can be attributed to the sensor’s lowest stiffness, which occurs at 0 PSI. The “softest” phantom does not cause significant deformation in the sensor, limiting its ability to provide a reliable response for stiffness estimation. To enhance deformation when the sensor is pressed against softer materials, the dome thickness should be reduced. In contrast, stiffness estimation for the other phantoms is relatively accurate and could be further improved through the application of data-driven algorithms.

## 4 Conclusion

In this paper, we have proposed a novel sensor based on abraded optical fibre, capable of dynamic force sensing. The results of our experiments show that the sensor’s force range increases with the internal dome pressure, while its sensitivity decreases. When applying pressures of 0 psi (0 kPa), 0.5 psi (3.45 kPa) and 1 psi (6.9 kPa), the maximum force measured at 5 mm/min was found to be 4.91 N, 6.54 N and 7.91 N respectively. Likewise, the maximum force recorded at a speed of 20 mm/min for pressures of 0 psi, 0.5 psi, and 1 psi was 5.02 N, 6.70 N, and 8.83 N, respectively. The maximum percentage errors calculated for hysteresis at indentation rates of 5 mm/min and 20 mm/min were 3.72% and 6.17%, respectively. We have also estimated the elastic modulus of 13 phantoms in a range of 7.00 kPa–165.32 kPa with an average RMSE of 3.23 kPa.

Moving forward, we plan on using learning methods to estimate the stiffness of different materials. We would also look into design improvements such as integrating a fabric mesh into the dome. This would restrict bulging and increase the force range of the sensor. For this sensor to become commercially viable, it would need to be miniaturised to 15 mm in diameter, so as to be able to pass through commercial trocar ports currently used in Minimally Invasive Surgery.

## Data Availability

The raw data supporting the conclusions of this article will be made available by the authors, without undue reservation.
